# Alkhurma Viral Hemorrhagic Fever Virus: Proposed Guidelines for Detection, Prevention, and Control in Saudi Arabia

**DOI:** 10.1371/journal.pntd.0001604

**Published:** 2012-07-31

**Authors:** Ziad A. Memish, Shamsudeen F. Fagbo, Abdullah M. Assiri, Pierre Rollin, Ali M. Zaki, Remi Charrel, Chris Mores, Adam MacNeil

**Affiliations:** 1 Public Health Directorate, Ministry of Health, Riyadh, Kingdom of Saudi Arabia; 2 Viral Special Pathogens Branch, Centers for Disease Control and Prevention, Atlanta, Georgia, United States of America; 3 Microbiology Laboratory, Doctor Soliman Fakeeh Hospital, Jeddah, Kingdom of Saudi Arabia; 4 Unite des Virus Emergents UMR190 “Emergence des Pathologies Virales″, Faculté de Médecine, Marseille, France; 5 Department of Pathobiological Sciences, School of Veterinary Medicine, Louisiana State University, Baton Rouge, Louisiana, United States of America; 6 College of Medicine, Alfaisal University, Riyadh, Kingdom of Saudi Arabia; Tulane School of Public Health and Tropical Medicine, United States of America

## Introduction

Alkhurma viral hemorrhagic fever virus (AHFV) is a tick-borne flavivirus described in Saudi Arabia and recently implicated in outbreaks of febrile illness associated with hemorrhagic and neurological manifestations. To facilitate an evidence-based approach to the public health challenges posed by this emerging pathogen, the Saudi Arabian Ministry of Health (MOH) convened a technical consultation of experts in the field of arboviral diseases on January 31, 2010, in Riyadh. The ensuing recommendations provide guidelines that should advance the clinical recognition, management, and prevention of AHFV infection. Areas requiring further research are also identified.

## The Virus

The AHFV was first isolated in 1995 from a 32-year-old male butcher that had acute, fatal viral hemorrhagic fever. It was confirmed a flavivirus by using a broadly reactive flavivirus monoclonal antibody, 4G2: this antibody reacts with dengue, yellow fever, West Nile and Alkhurma viruses. Further studies at the United States Centers for Disease Control and Prevention (CDC) affirmed it was a novel flavivirus related to the Kyasanur Forest disease virus (KFDV), a member of the mammalian tick-borne virus subgroup of the genus *Flavivirus*, family Flaviviridae, that causes hemorrhagic fever in Karnataka State, India [1]. It was named Alkhurma after the village from which the sheep was sourced [2] and has been approved by the International Committee for Taxonomy of Viruses. Analysis of its wholly sequenced genome reaffirmed that it is a tick-borne flavivirus and a genetic variant of KFDV [3].

## Clinical Manifestations

Analysis of the first set of confirmed Alkhurma hemorrhagic fever (AHF) cases suggested a pattern characterized by severe to fatal clinical outcome with a case fatality rate approaching 30% [2]. Clinical and laboratory characteristics of the earliest virologically confirmed AHF cases (*n* = 16) included non-specific febrile episodes, leucopenia, thrombocytopenia, and elevated serum liver enzymes, with some patients having hemorrhagic and/or neurologic manifestations [4]. This initial description, based on these few cases, should evolve for one or more reasons. Firstly, the preliminary data set may be biased as only the most severe cases were detected: recent data [5] suggest that mild or even asymptomatic AHF do occur. For example, in 2010, only 81 cases were documented nationwide, with two fatalities. Secondly, the present level of diagnostic capability may not accurately recognize infections due to AHFV or other endemic flaviviruses: as flaviviruses are notoriously cross reactive, serology-only diagnostics may not accurately pinpoint the flaviviral aetiologies in clinical cases of viral hemorrhagic fever. This has been observed in areas where dengue and AHF are co-endemic. All these factors contribute to the perceived low index of suspicion that persists amongst clinicians in some parts of the country.

## Vector(s) and Reservoir(s)

There is strong virological, entomological, epidemiological, and phylogenetic evidence that the AHFV is a tick-borne flavivirus [3], [6], [7]. Based on limited data, vectors/reservoirs of AHFV presently include the soft tick *Ornithodoros savignyi* and the hard tick *Hyalomma dromedarii* [6], [8]; AHFV has been isolated from these two species, which are also endemic in neighboring countries [9]. Although AHFV has not been detected in animals, livestock that are often extensively ectoparasitized by these ticks have been epidemiologically linked with acute AHF in the Makkah and Najran regions [1], [5]. Such cases have been mainly associated with camels and sheep. However, as with other tick-borne viruses, these animals may only be acting as hosts for transmission between ticks as well as tick amplification.

## Challenges

Tackling a relatively new infection with a complex, multi-host transmission cycle can be daunting. Moreover, given the previously mentioned premises that stoked public health concerns, these constitute challenges that need to be urgently met.

Approaching these challenges from a one-health and multidisciplinary platform, the Saudi Arabian MOH convened a technical consultation involving experts in the field of arbovirology, emerging pathogen epidemiology, and entomology from the US and France. Other participating governmental agencies included the Ministry of Agriculture, Ministry of Municipalities, and the Saudi Wildlife Authority. The plenary session provided an update on the epidemiology, clinical characteristics, potential vector, and related livestock/wildlife/human disease interface. Subsequently, five focus groups representing various subject areas were assembled (Box 1). Issues relating to the various domains and the consensus recommendations reached are presented below. Opportunities exist to integrate these recommendations with presently existing vector-borne disease programs (e.g., RVF and dengue).

**Box 1. Focus Groups for AHF Workshop**

- Case definition/epidemiologic studies
- Vector/reservoir identification
- Health education and awareness
- Laboratory diagnosis
- Infection control

Although AHF has only been reported in the Arabian Peninsula and Egypt [10], it can be expected to have a wider geographical spread. This meeting thus provided guidance for further research that should enhance the clinical recognition, management, and prevention of AHF. Lastly, expected research outcomes should support future prevention efforts while incorporating similar work done on other related tick-borne flaviviruses [11], [12].

## Case Definition and Epidemiologic Studies

One of the problems limiting accurate recognition of AHF is the lack of a standardized surveillance case definition sensitive enough to identify most suspect cases, thereby triggering early diagnostic testing and further investigations. An earlier case report [13] describing eight IgG-only positive cases as laboratory confirmed AHF exemplifies this problem. Paired sera were not tested and consideration was not given to potential secondary or sequential infection with another flavivirus. It further highlights the need for a standard case definition incorporating validated flaviviral diagnostics found in the literature [14]. Diagnostic challenges in confirming the causal flavivirus in clinical cases from regions where several flaviviruses are co-endemic is not something new: in the US, the first case of West Nile Virus (WNV) was initially misdiagnosed as St. Louis encephalitis (SLE) virus infection by the CDC [15]. The situation is exacerbated when these viruses cause infections associated with nonspecific clinical syndromes.

Locally, lack of familiarity with tick-borne disease ecology may be adversely limiting clinical recognition of AHFV infection as well as other tick-borne pathogens [16]. For cases that are tick transmitted, as with other tick-borne diseases [17], [18], tick exposure recall may not be possible in all cases of AHF. Recognition of tick bite may be affected by tick size. Lastly, recalling *Ornithodoros* spp. tick bites could be challenging, as these ticks do not normally remain attached for more than an hour. The recommendations in Box 2 are designed to address these issues.

**Box 2. Recommendations – Case Definition and Epidemiologic Studies**

**1. Clinical case definition (human cases):**

*Suspected*: Case meets the clinical AND exposure criteria.

*Probable*: suspected case with clinical laboratory data (e.g., thrombocytopenia, leucopenia, elevation of liver enzymes, elevated CPK or LDH) and IgM detected by capture ELISA

*Confirmed*: Probable case AND laboratory criteria listed below

Clinical criteria:

a. Unexplained acute febrile illness (fever >38°C) with one of the three following features:

i Hemorrhagic manifestations not related to injury (bleeding under the skin, in internal organs or from body orifices; and positive tourniquet test)
ii Liver involvement (jaundice, hepatomegaly)
iii Neurological involvement (severe headache, altered mental status, and/or seizures)

Laboratory criteria:

a. One or more of the following laboratory findings:

iv AHFV RNA detected by real-time or conventional RT-PCR
v Virus isolation/identification using cell culture or suckling mice
vi Four-fold antibody (IgG) rise in paired serum samples using ELISA or IFA
vii Neutralization test—preferably plaque reduction for paired sera

Exposure:

b. One or more of the following exposures before onset of symptoms:

viii Recent contact with animal, blood, or other animal products
ix Recent exposure to or bite by tick
x Contact with blood or body fluid from a confirmed human case
xi Work in a laboratory that handles AHFV specimens/isolates

**2. Epidemiologic studies.**

- Standardised forms should be used to capture all relevant clinical, epidemiological, and laboratory data (clinical history form, epidemiological investigation form, and laboratory requisition form). A unique single case identification number, assigned to each suspect case and printed on all forms and diagnostic samples, should link case and laboratory data.
- To determine an initial point prevalence of AHF, healthy blood donors should be screened for AHFV-reactive antibodies and positive results subjected to confirmatory tests. Samples should be drawn from areas presently with (Makkah/Najran regions) or without (Eastern/Riyadh regions) reports of AHF. This would provide prevalence estimates to determine the sample size for a broader, similar study.
- Diagnostic confirmation of exposure and prevalence studies in livestock (in particular camels and sheep) in the field as well as abattoirs is recommended in order to evaluate the epidemiologic association between these animals and previous human cases. We could thus improve our knowledge of sources of infection and indicators of exposure risks in the environment.
- Given that the findings from recommendation 2c (above) could indicate that the association between human cases and livestock exposures are not spurious, a program of active surveillance of livestock, especially when abnormal patterns of morbidity (e.g., spontaneous abortion) or mortality occur, is recommended. This could also help determine whether AHFV infections in livestock are asymptomatic, or are otherwise under- or mis-diagnosed. Such efforts can be incorporated into a similar pre-existing system for RVF, thereby enhancing livestock pathogen surveillance in the region. For such surveillance, hemagglutination inhibition (HI) or ELISA can be used.
- Additionally, the effects of AHFV infection in appropriate lab models could be investigated under high containment.
- The presence and duration of viable virus in unpasteurised milk under various physico-chemical conditions should be determined. Milk from experimentally infected lactating animals should be evaluated. The same applies to milk and sera from animals epidemiologically linked to laboratory confirmed human cases.

## Vector and Reservoir Identification

Apart from the fore-mentioned ticks, other potential vectors exist, as a number of medically important ticks have been described [9]. This includes *Haemaphysalis sulcata*, which is similar to *Haemaphysalis spinigera*, the main vector of KFDV in India [19]. These ticks, some already associated with Crimean-Congo hemorrhagic fever (CCHF) and Kadam ecology [20], [21], have a complex multi-host life cycle. AHFV RNA and virus were detected in the soft tick (*Ornithodoros savignyi*) associated with camel resting place in Eastern Jeddah [6] and Najran market [8]. *O. savignyi* is widely distributed in the Arabian Peninsula and can survive in a dormant state for extended periods of time [9]. More studies are needed to confirm the vector status of these ticks as well as understand the intra-tick AHFV replication dynamics; relevant studies have been recommended in Box 3. The role of other arthropods (mosquitoes, Culicoides, sand flies, etc.) in AHFV ecology remains unknown, and a suggested mosquito-transmission hypothesis [12] is unsubstantiated.

**Box 3. Recommendations – Vector and Reservoir Identification**

1. Ticks from tick-infested livestock should be tested for AHFV. To sample adult hard ticks, assuming a 1% infection rate and tested in pools of five or fewer individual ticks, it is suggested that at least 250 ticks per sample site per sample time point with preferably no more than one pool per tick species per animal.
2. Studies confirming the vectorial capability of *Ornithodoros savignyi* and *Hyalomma dromedarii* and other potential vectors are required. The experimental infection/inoculation of ticks at different stages of development using suitable animal models in appropriate arthropod containment level facilities is recommended.
3. Well focused, multidisciplinary ecological studies elucidating transmission patterns in various ecotypes are recommended: data elicited should guide interventions aimed at controlling AHFV vectors. These studies should reveal the fauna hosting immature stages of potential AHFV vectors.
4. Acaricide sensitivity, resistance, and environmental effects should be regularly monitored.
5. Of lesser priority, other potential arthropod vectors (mosquitoes, midges, sand flies, etc.) could be tested for AHFV and evaluated for vector competency.
6. The possible role of bats and birds in AHFV ecology should be evaluated, serologically and experimentally.

Although livestock have been linked with human infection, they may not be the reservoir hosts of AHFV. The putative vectors (all stages of *O. savignyi* and the earlier life stages of *H. dromedarii*) are not usually found on livestock. The different life stages of these ticks have a broad host range: apart from livestock, they parasitize a wide range of local fauna [22], [23]. The known ecology of KFDV may provide insight as to what to expect for its genetic variant, AHFV. The first outbreak of KFDV in Karnataka coincided with fatalities in monkeys from the nearby Kyasanur forest: KFDV was isolated from these monkeys and associated *Haemaphysalis* ticks [24]. The role of various life stages of *Haemaphysalis* ticks, rodents, and other small mammals native to this forest in the ecology of KFDV has been documented [25], [26]. Rodent-to-human transmission of KFDV has also been suggested [18].

## Health Education and Awareness

Presently, there are no antivirals or vaccines to treat or prevent AHF, but cases are symptomatically managed. Consequently, adequate AHF awareness material should be provided for the public, especially for those at high risk (see Box 4). Based on published [1], [5] and internal MOH data, high-risk individuals include slaughterhouse workers, butchers, and shepherds. Those drinking raw, unpasteurised milk are also thought to be at risk [2]. Milk-borne transmission of tick-borne encephalitis virus, another mammalian tick-borne flavivirus, has been documented [27]. Seasonally, people are at risk when they are involved in selling or slaughtering animals for the yearly pilgrimage/sacrifice (Hajj/Eid ul Adha) as well as cutting up meat from infected animals.

**Box 4. Recommendations – Health Education and Awareness**

1. Tick-avoiding measures, including the use of repellents and protective clothing and avoiding tick-infested areas or animals, are recommended.
2. Methodical and supervised chemical control of ticks is recommended where applicable.
3. Use of appropriate personal protective equipments when handling animals or animal products in farms, houses and slaughterhouses.
4. Increase awareness about the disease, mode of transmission, and arthropod avoidance behavior. By leveraging with ongoing RVF prevention programs, it is possible to reach out to at-risk persons as well as the general public.

## Laboratory Diagnosis

Prompt, accurate laboratory recognition of AHFV infection enables appropriate clinical management, infection control, and public health intervention.

However, challenges abound. The first challenge emanates from the antigenic composition of flaviviruses and established serologically relatedness of flaviviruses [28], [29]. Other flaviviruses are known or suspected to be locally endemic [30]. Additionally, other flaviviruses such as Japanese encephalitis virus may be imported by migrant workers and remain undetected. The accurate interpretation of AHF serological tests may thus be affected by possible flaviviral cross reaction. This challenge is further demonstrated by the first report of AHF outside the Kingdom of Saudi Arabia [10]. In that report, the first patient was positive for West Nile Virus and dengue virus IgM. These initial results were ruled out as false positives by sequencing the amplicon product of a RT-PCR run using a genus-specific flavivirus primer. The sequence showed a high homology with the AHFV sequence AF331718 deposited in GenBank. Secondly, panel deliberations and MOH observations indicate that accurate recognition of AHF has been affected by inappropriate sample labeling, storage, and shipping. Thirdly, tests currently used to test for AHFV infection remain unvalidated. Lastly, the viremic phase remains undetermined and the proficiency of available tests to detect infection in various biological specimens taken at different stages of illness is unknown. These concerns have been addressed in the recommendations detailed in Box 5.

**Box 5. Recommendations – Laboratory Diagnosis**

1. Four national laboratories will be equipped with biosafety and technical requirements to handle the specimens.
2. The AHFV kit will include adhesive and biohazard labels, laboratory and clinical forms, and three 5-ml tubes: gel tube for serology (avoids hemolysis and permits storage at −20°C or −80°C if necessary); and EDTA and heparin tubes for RT-PCR and virus isolation, respectively. However, ELISA may be performed on sera, plasma, and poorly preserved (hemolysed) blood. Pre-printed adhesive tags with unique ID number should be included in the ready-to-use collection kit
3. Lab form should include the following:
  - Patient's name with barcode label
  - Patient number (hospital number) and specimen number
  - GPS coordinates of patient's location.
  - Time/date sampled
  - Time/date received at the testing lab
4. Specimens should be properly labeled and immediately shipped in dry ice (−78.5°C) to the designated lab. If this is not possible, ship on wet ice (+4°C). If a specimen is to be delayed for more than 24 hours, store at −80°C (plain tubes with gel should be centrifuged before storage; this is not required for EDTA or heparin tubes).
5. It is recommended that current tests—ELISAs, IFA, and RT-PCR—are validated.
6. Additionally, the proficiency of available tests to detect AHFV infection in different biological samples taken at various stages of acute illness and convalescence should be evaluated.
7. Efforts should be exerted to improve tests or design new ones (ELISAs, Western blots) that can be deployed in a non-high-containment lab situation. Priority should be given to robust and validated RT-PCR and ELISA platforms taking into cognizance other endemic flaviviruses. The expected increased diagnostic ability nationwide should provide more accurate data on AHFV geographical prevalence. Additionally, new ELISAs using recombinant protein technology to target non-cross reactive epitopes of AHFV envelope protein should increase specificity while significantly reducing cross reactivity.

## Infection Control

Though AHFV is a genetic variant of KFDV, there is a lack of robust local risk assessments to guide laboratory manipulation of suspected samples. However, there have been no reports of person -to-person (patient contacts/health care workers) transmission [31]. However, the hemorrhagic nature of a subset of acute AHF cases dictates that optimal infection control measures are maintained in clinical settings. Suggestions are provided in Box 6.

**Box 6. Recommendations – Infection Control**

1. It is recommended that a risk assessment to guide laboratory manipulation of suspected clinical samples/isolates is carried out.
2. Efforts to increase awareness about AHF epidemiology amongst health care workers (HCWs) should be aligned with the medical curricula and continuing medical education programs.
3. The orientation of new HCWs should incorporate training on endemic arboviral and tick-borne zoonoses such as AHF.
4. In addition to standard infection control practices, local infection control guidelines stipulate that patients with suspected or confirmed hemorrhagic fever be admitted to an isolation room with negative pressure or an appropriate ward if a designated isolation unit is not available.
5. HCWs in contact with confirmed AHF patients should be monitored for possible seroconversion or clinical signs.

## Conclusion

The challenges posed by AHF were discussed during the course of this technical consultation. Improved diagnostics, along with a well disseminated and applied case definition, should provide a more accurate picture of AHF prevalence in Saudi Arabia and the neighboring regions.
